# A study of clinical and economic burden of surgical site infection in patients undergoing caesarian section at a tertiary care teaching hospital in India

**DOI:** 10.1371/journal.pone.0269530

**Published:** 2022-06-03

**Authors:** Shilpa Hirani, Niyati A. Trivedi, Janki Chauhan, Yash Chauhan

**Affiliations:** Department of Pharmacology, Medical College & SSG Hospital, Vadodara, Gujarat, India; University of Waterloo, CANADA

## Abstract

**Background:**

Caesarian section is one of the most commonly performed surgeries in India. Determination of the incidence as well as the clinical and financial burden of post caesarian surgical site infection (SSI), is of critical importance for all the stakeholders for rational and fair allocation of resources.

**Methods:**

This study was a prospective observational case-control study. The mean direct and indirect cost of treatment for the cases were compared with the control patients. An unpaired t-test was used to compare the mean between the two groups.

**Results:**

Out of 2024 patients, who underwent caesarian section during the study period, 114 had acquired incisional surgical site infection (ISSI), with the infection incidence being 5.63%. The total cost of illness due to post caesarian ISSI was almost three times higher compared to the non-infected matched control group. (P<0.0001). An average length of hospital stay in the ISSI patient group was 10 days longer than that in the control group (P<0.0001) and importantly total length of antimicrobial therapy(LOT) in patients with ISSI was also almost three times higher than the control group (P<0.0001).

**Conclusion:**

The development of post caesarian SSI imposes a significant clinical as well as a financial burden. The study highlights the necessity of taking effective preventive measures to decrease the incidence of SSI.

## Introduction

Surgical Site Infection (SSI) is used to encompass the surgical wound and infections involving the body cavity, organs, which may or may not be associated with implants or prosthetic devices [1].

SSI is the most frequent type of hospital-acquired infection (HAI) in low-middle income countries (LMICs) and affects up to one-third of patients who have undergone a surgical procedure. While SSI incidence is much lower in high-income countries (HIC), however, it remains to be the second most frequent type of HAI [2].

SSIs can have major implications for patients, hospitals, and society; they increase morbidity as well as mortality [3], and as such may contribute to the burden on society in terms of healthcare costs (economic burden) and loss of years experienced in full health. SSIs may also result in excess healthcare utilization and costs. Previous studies have shown that costs can double, triple, or even increase six-fold for patients with an SSI compared with patients without an SSI [4–6], depending on the type of surgery, health care setting, and type of infection.

Though comprehensive, evidence-based guidelines exist for the prevention of SSI and many studies have confirmed reduced rates of wound sepsis by the successful implementation of the same [7–10], however, overall there is gross non-compliance and lack of initiative from the stakeholders on the implementation of such best care practices.

To our knowledge, only a few hospitals in India have allocated adequate resources to perform regular surveillance on SSI and for effective implementation of prevention practices.

A critical step in convincing the health care authorities to allocate scarce resources to infection control programme is to demonstrate that these interventions will not only reduce the rate of infection but will also result in savings that exceed the cost of preventive strategies [11,12]. Thus a better understanding of the financial burden of SSI would help to make decisions on the economic benefit of greater investment in evidence-based interventions to prevent them.

A cost of illness (COI) study quantifies how much society is spending on a particular disease and represents the cost burden averted if the disease was eradicated. Due to different international healthcare systems and healthcare financial systems, costs calculated in HICs cannot be translated directly to LMICs settings. Hence, to obtain useful estimates, country-specific data and surgery type-specific data should be assessed for our country.

As caesarian section is one of the most commonly performed surgeries in India, and itself imposes a great economic impact on the health care system, understanding the additional cost burden imposed by post caesarian SSI, will help to generate relevant evidence to take decision on the prioritization of resource allocation for implementation of an intervention for effective preventive and control strategies.

The present study compares the main direct and indirect costs of treatment for matched patients undergoing caesarian section who did or did not acquire a SSI.”

## Material and methods

### Study setting

This prospective observational study was performed at a 1500 bedded tertiary care teaching hospital in Gujarat state, India in the inpatient department of Obstetrics at S.S.G. Hospital, Baroda. It is a state government-funded hospital, so the cost of treatment is borne by the state government.

### Study population

Cases were all patients admitted in wards of Obstetrics department who underwent cesarean section at S.S.G. Hospital, Baroda, during the period of July 2019 to March 2020, irrespective of indication for cesarean, type of operation (elective or emergency), their parity, or amniotic membrane status, who satisfied the criteria of incisional surgical site infection(ISSI) according to CDC guidelines^13^, and had given written informed consent.

ISSI was identified by using the following criteria, either singly or in combination according to CDC guidelines [13].

Date of event occurs within 30 days after the cesarean section (where day 1 = the procedure date) AND involves only skin and subcutaneous tissue of the incision AND patient has at least one of the following:

- purulent drainage from the superficial incision.
- organism(s) identified from an aseptically-obtained specimen from the superficial incision or subcutaneous tissue by a culture or nonculture based microbiologic testing method which is performed for purposes of clinical diagnosis or treatment.
- superficial incision that is deliberately opened by a surgeon, physician or physician designee and culture or non-culture based testing of the superficial incision or subcutaneous tissue is not performed AND patient has at least one of the following signs or symptoms: localized pain or tenderness; localized swelling; erythema; or heat.
- diagnosis of a superficial Incisional SSI by a physician or physician designee.

In order to calculate differential costs, ISSI patients were compared to matched control patients. Controls were selected randomly among the patients who underwent cesarean section during the same period but did not develop infection. They were individually matched to cases on key attributes such as age, and co morbidities including ASA score.

### Data collection procedure

All patients operated for cesarean section at the study institute were screened and data was collected of patients who developed ISSI and their matching controls. Demographic and clinical details of such patients were obtained from the case records of the patient. The laboratory reports, as well as microbiology report of specimen (e.g. wound swab, pus or tissue) obtained from patients, were screened on daily basis. All such patients were followed up on daily basis until they were discharged from the hospital and the duration of hospital stay was noted.

Outcome and economic analysis were limited to ISSIs occurring during admission or on readmission, with the latter aggregated with those of the original admission to form a single patient episode. Only the first episode of the ISSI was included in the study.

### Various cost included in the study

The costs of illness considered in the study included direct as well as indirect costs. The intangible cost has not been taken into consideration in the study.

The direct cost of treatment included the cost of antimicrobial agents and other concomitant medication used, cost per bed days, and cost of diagnostics procedure during hospitalization.

The indirect cost of treatment included travel cost to the hospital, boarding, and lodging as well as loss of productivity.

### Data/Definition used for the calculation of various costs included in the study

**1**. **Direct cost**:

1.1 Direct medical cost

The cost of the antimicrobials and other concomitant medication used during hospitalization was obtained from the purchase department of our hospital setting.

1.2 Direct non-medical cost

For calculation of cost per inpatient stay (I.e. cost per bed-day) which included the human resource, capital cost, and material cost were taken into consideration based on the study by Chatterjee S *et al* [14] and appropriate inflation rate for 2019–2020 was applied.

1.3 Diagnostic cost: included the cost of lab investigations carried out during inpatient stay including microbiological investigations. As the cost of treatment, as well as diagnostics, is borne by the hospital, only the material cost has been calculated here.

**2. Indirect cost**:

Indirect cost is the money spent for purposes other than treatment but indirectly related to treatment like travel, boarding, and lodging and loss of income due to absence from work due to the illness.

2.1 Travel cost: As patients used different modes of travel, to reach the institute, which included, on foot, use of private/personal vehicle, or public transport. To maintain uniformity, we calculated the charge/km as decided for reimbursement policy by the Government of India [15]. For the patients who developed ISSI, during their hospital admission, as well as for the control group, only one-time travel costs to and from the place of residence were calculated. However, for the patients who were readmitted for ISSI, travel cost for the patient and one accompanying person was calculated as per the number of follow up visits including the readmission.

2.2 Boarding and lodging cost: As almost all patients in the study were from the low socioeconomic background, could not afford the boarding and lodging facility for the accompanying person, during the period of hospital stay, on their own, and as many of them accepted the free boarding lodging services offered to them by different organizations, it was not possible to uniformly calculate the amount spent on the same. So to get an estimation of the same, we opted to use the average per day boarding & lodging cost calculated by another study conducted for a tertiary care teaching hospital, from the same tier of the city in which our city belongs [16].

2.3 Loss of productivity cost: We applied the wages / per day index for the loss of productivity cost for the accompanying person based on the Director of Labour, labour and employment department, Government of Gujarat website. (https://col.gujarat.gov.in/news.htm) [17].

The total cost of illness included the sum of direct as well as indirect costs.

### Data analysis

Statistical analyses was performed using the Graphpad Prism software (version 9.1.0(221)). The data are expressed as mean ± standard deviation (SD) or number (%). Cost data were also expressed in range (min-max). An unpaired two-tailed t-test was used for comparison of data between two groups. P<0.05 was considered as statistically significant.

For analysis of difference between proportions, fisher exact test (two tailed) was performed. P<0.05 was considered statistically significant.

The cost data were obtained and calculated in Indian rupees and later were converted to US dollar. Conversion rate 1 US $ = 74.88 INR was applied as on the day of analysis i.e. 9^th^ april 2021 (Reserve Bank of India,India’s central bank [Internet]. Available from: https://www.rbi.org.in/Scripts/BS_DisplayReferenceRate.aspx. last assessed on 09-04-2021).

## Results

Out of 2024 patients who underwent cesarean section during the period of July 2019 to March 2020, at the department obstetrics & gynecology, at our tertiary care teaching hospital, 114 patients (5.63%) developed incisional surgical site infection secondary to cesarean section. Mean no. of days(±SD) from date of surgery to diagnosis of wound infection (ISSI) was 6.97 ± 2.7 days with a range of 4 to 14 Days. (Day of surgery is considered as day 1). Out of which 71% patients were diagnosed to have ISSI during their hospital stay, while 28.95% patients were readmitted for ISSI later. In case of readmission, length of stay (LOS) was aggregated with those of the original admission to form a single patient episode.

There was no significant age difference between the control and ISSI patients. The mean preoperative LOS, as well as number of surgical antimicrobial prophylaxis prescribed in patients with ISSI, was comparable to the control group. The percentage of emergency cesarean was significantly higher in ISSI patients compared to the control patients. (P<0.0001). None of the patients either in the control or ISSI group had Diabetes Mellitus. (Table 1).

**Table 1:** Comparative characteristics of patients with post caesarian ISSI and control group.

Characteristics	Control Group (N = 114)	Patients With ISSI (N = 114)	P
Age	26.12(5.69)	24.89(4.7)	0.07
Preoperative LOS	1.41(0.93)	1.13(1.00)	0.029
Total LOS	5.33(0.65)	14.78(3.33)	<0.0001
Number Of AMA Prescribed As SAP	2.58(0.52)	2.14(0.67)	<0.0001
Total AMA LOT	4(0.91)	15.14(2.93)	<0.0001
Total AMA DOT	10.33(3.82)	28(6.19)	<0.0001
Type Of Surgery	Elective(33%) Emergency(67%)	Elective(8.7%) Emergency(91.2%)	<0.0001

Table 1: Data are presented as mean(SD) or percentage.

AMA: Antimicrobial agents; SAP: Surgical antimicrobial prophylaxis; LOS; Length of hospital stay; LOT: Length of (antimicrobial) Therapy; DOT: Days of (antimicrobial) therapy.

There was a significant difference in the total hospital LOS between the ISSI and the control group. Mean LOS in patients with ISSI was 14.78 +3.33 days which was almost three times higher compared to the control group. (P<0.0001) (Table 1).

### Antimicrobial exposure

As a routine practice of the institute, microbiological diagnostic testing is done for all the patients, suspected to have SSI.

Out of 114 patients with ISSI, in 52 (46%) patients one or more microorganisms were isolated from their clinical specimen. In five specimens more than one type of microorganism was isolated. The most common organism isolated was *Acinetobacter spp*. (37%) followed by *Klebsiella spp*. (25%) and *E*. *coli spp*. (21%). In one patient the microbiology testing was done twice due to no response to treatment.

In total 52 clinical specimens, 57 microorganisms were isolated and they were tested against a total of 27 different antimicrobial agents (AMAs) for their sensitivity. Each isolate was tested against an average of 7.63±0.90 AMAs, out of which it was resistant to 5.23±1.52 AMAs. The majority of the microorganisms showed > 50% resistance to most of the antimicrobials tested except for high-end or reserved AMAs (i.e. Meropenem; Colistin, Linezolid, Polymixin-B, and Tigecycline).

All 114 patients who developed ISSI, were given three or more AMAs during their course of treatment. Out of which 57% were prescribed four AMAs, 25% were prescribed 5 AMAs, and 13% were prescribed six or more than six AMAs during their course of treatment.

Days of therapy (DOT) and Length of therapy (LOT) are commonly used antimicrobial usage metrics [18]. DOT is defined as the number of days that a patient receives an antimicrobial agent (regardless of dose). Any dose of an antibiotic that is received during a 24- hour period represents 1 DOT. The DOT for a given patient on multiple antibiotics will be the sum of DOT for each antibiotic that the patient is receiving [18].

LOT is defined as the number of days that a patient receives systemic antimicrobial agents, irrespective of the number of different drugs. Therefore, LOT will be lower than or equal to DOT because each antibiotic received is its own DOT [18].

The ratio of DOT/LOT may be useful as a benchmarking proxy for the frequency of combination antibiotic therapy vs. monotherapy. That is, ratio = 1, identifies monotherapy; ratio > 1 identifies combination therapy.

In the ISSI group, the Mean DOT (Days of therapy) of AMA was 28 ± 6.19 days with a range of 18 to 48 days. In contrast, the mean DOT of AMA therapy for the control group was 10.33 ±3.82 (P<0.0001).

Mean LOT (Length of therapy) of AMA in the ISSI group was 15.14 ± 2.93 days with a range of 10 to 25 days. While in the control group it was 4 ±0.91 (P<0.0001).

### Cost of illness due to ISSI

**Direct cost**

**1.1 Cost of antimicrobial prescribing**:

The total cost of antimicrobial prescribing in patients with ISSI was significantly higher compared to the control group. While for the patients in the control group, the average number of AMAs prescribed was restricted to 2.58± 0.52 AMAs, prescribed for four days as surgical antimicrobial prophylaxis, however, for patients with ISSI, it was 4.41±0.95 AMAs, prescribed for 15.14± 2.93 days. Which resulted in nearly 10 days of additional AMA exposure and 11 times higher cost of antimicrobial treatment. (P<0.0001). The average cost of AMA in the control group was 2.80±0.86 $, with a range of 1.83–3.98 $, (INR 209.75±64.27 with a range of 137.16 to 297.60); while the same cost in patients with ISSI was 30.75 ±20.02 $ with the range of 6.54–119.36 $ (INR 2302.46± 1498.65 with the range of 489.96 to 8937.36) (Table 2).

**Table 2:** Cost of treatment (in USD) in control and cases with post cesarean ISSI.

	Cost of AMA	Cost of concomitant medication	Diagnostic cost	Cost per hospital days	Capital loss due to loss of productivity	Travel cost	Lodging and boarding	Cost of treatment per patient
ISSI	30.75 (20.02)	2.18 (2.15)	2.48 (0.43)	181.40 (36.12)	76.28 (15.19)	6.81 (5.01)	63.22 (20.11)	363.89 (86.32)
Range	6.54–119.36	0.45–9.45	2.14–3.87	97.24–345.75	40.89–145.39	2.30–40.39	38.13–146.17	222.96–769.40
Control	2.80(0.86)	0.83(0.77)	0.85(0.08)	57.63(7.04)	24.23(2.96)	4.88(2.73)	33.88(4.14)	124.90 (14.56)
Range	1.83–3.98	0.36–2.49	0.80–1	43.22–64.83	18.16–27.26	1.15–9.62	25.42–38.13	96.80–144.33)
*P*	<0.0001	<0.0001	<0.0001	<0.0001	<0.0001	<0.001	<0.0001	<0.0001

Table 2: *Data are expressed as mean(SD) and range(min-max)*. *Two tailed un- paired t test is used for comparisons between two groups*. P*<0*.*05 is considered statistically significant*.

Cost was calculated in Indian rupees and later converted to USD as per the prevailing conversion rate.

**1.2 Cost of concomitant medication**:

The average cost of the concomitant treatment in patients with ISSI was 2.18 ± 2.15 $ (INR 218.08±161.30) which was significantly higher compared to control (0.83 ± 0.77 $ or INR 61.98± 57.49). (P<0.0001) (Table 2).

**1.3. Hospital cost per bed days**:

As the patients with ISSI had nearly four times higher total length of hospitalization, the average total cost per bed days in patients with ISSI was 181.40 ±13 $, (INR 582.68± 2704.59), which was significantly higher compared to 57.63 ± 7.04 $ (INR 4314.667 ±526.93) for control patients. (P<0.0001) (Table 2).

**1.4. Diagnostic cost**:

Average diagnostic cost per patient was 2.48±0.43 $ (INR 185.70± 32.14) in patients with ISSI compared to 0.85±0.08 $ (INR 63.89± 6.01) in control patients (Table 2).

**2. Indirect cost**:

**2.1 Travel cost**:

Only one time to and from travel costs were calculated for the control as well as patients who had their episode of ISSI while in the hospital, and an actual number of follow-up visits were considered for readmissions. Mean travel cost in the ISSI group was 6.81 ± 5.01 $ compared to 4.88± 2.73 $ in the control group, (P<0.001)

**2.2 Cost of lodging and boarding** for the patient’s relatives:

The cost of lodging and boarding was calculated based on the study performed in the similar tier of city in India. Due to the longer length of hospital stay, the parallel cost of lodging and boarding in patients with ISSI was significantly higher compared to the control group (P<0.0001) (Table 2).

**2.3 Cost to loss of wages**:

72.80% of the women were working as semiskilled laborers before they underwent cesarean section at our hospital. However, as per the local custom, postpartum women would otherwise take at least a few days of work-leave, their loss of wages has not been considered here.

All our patients were from poor socio-economic background, who could not afford a care taker for the new borns. And moreover, as per the local Indian custom, for the initial few months, usually the new born baby is taken care of by the close first or second degree female relative of either spouse. So that cost has also not been taken into consideration.

So cost to loss of wages of only the accompanying person have been calculated. The average loss of productivity of the accompanying person among patients with ISSI was 76.28 ± 15.19 $ (INR 5711.78± 1137.33) compared to 24.23 ± 2.96 (INR 1814.4± 221.58) for the control group (Table 2).

In our study, the total cost of treatment for patients with ISSI was 41,521 USD (INR 31,06,010), which was 2.91 i.e. almost three times higher than the equally sized control group. With per patient average total cost of treatment in the ISSI group was 363.89 ± 86.32 $ with a range of 222.96–769.40 $ (INR 27,246.43±6463) compared to 124.90 ±14.56 $ (INR 9351.84± 1090.40) in the control group (Table 2).

At our settings, the direct and indirect cost of treatment for all the patients, except for the financial impact caused by loss of productivity, as well as the travel cost, was borne by the government-funded hospital.

Subgroup analysis based on the presence of Co morbidities was initially planned, but couldn’t be done because 1) the number of patients with pre-eclampsia was too small and 2) almost all the patients with ISSI had anemia, and the same for the controls who were matched on several characteristics including anemia.

## Discussion

While advances have been made in infection control practices, SSIs remain a substantial cause of morbidity, prolonged hospitalization, and death. SSI is associated with a mortality rate of 3%, and 75% of SSI-associated deaths are directly attributable to SSI [13].

SSIs not only delay recovery, and prolong hospitalization; they are also responsible for the significant psychological distress and economic burden to the society.

In our study, the incidence of developing post-cesarean section ISSI was 5.63%. The reported rate of SSIs varies considerably amongst the studies conducted at different centers around the world ranging from 2.10% in Kuwait [19] to a rate as high as 48.20% in a resource-limited Tanzanian hospital [20]. The variation in SSI incidence may reflect differences in population characteristics, risk factors, type of surgery, peri-operative practices, and post-discharge infection surveillance [19].

The majority of the patients (78%) in our study underwent cesarean section because of fetal distress. The percentage of patients undergoing emergency cesarean section was higher in the ISSI patient group compared to the control group. Generally, patients undergoing emergency cesarean are at higher risk of infections. This may be because of inadequate preparation time owing to maternal or fetal threat [21].

In the present study, the Mean ± SD number of days from the date of surgery to diagnosis of wound infection (ISSI) was 6.97(2.7) days. *Acinetobacter spp*. was the most common isolated microorganism (37%) followed by *Klebsiella spp*. (25%), *E*. *coli spp*. (21%) and Methicillin-resistant *Staphylococcus Aureus* (7%).

All isolates obtained from the ISSI patients were multidrug-resistant organisms. Each isolate was tested against an average of 7.63±0.90 AMAs, out of which it was resistant to 5.23±1.52 AMAs.

The average antimicrobial length of therapy (LOT) in the ISSI patient group was 15.14± 2.93 days, which was almost four times higher than the control group. Even for the control group, the LOT for antimicrobial therapy in the present study is 4± 0.91 days, while most of the standard treatment guidelines recommend a single dose of surgical antimicrobial prophylaxis within 60 minutes before incision for most of the instances [22,23].

Indiscriminate use of AMAs along with inadequate IPC practices creates a vicious cycle, with the higher number of multidrug-resistant isolates, which prolongs the duration of AMA therapy as well as length of hospital stay, which further adds to the risk of antimicrobial resistance.

Moreover, prolonged AMA therapy leads to a significant increase in the cost of AMA prescribing along with other direct and indirect costs of treatment due to prolonged length of hospitalization.

In our study, there is a significant increase in the length of hospitalization in patients with ISSI. Patients with ISSI were estimated to have an average hospital LOS of 10 days longer than the control group. This result is consistent with previous studies; however, the days of prolongation in hospital LOS varied among studies [3,24,25].

Development of SSI will delay patients’ recovery from surgery, resulting in extended hospital LOS. Hospital LOS can be considered as one of the most important predictors of COI study, as the prolongation of LOS not only increase the direct and indirect cost of treatment for the current clinical condition, furthermore, occupied hospital beds also compromise the availability of beds to other potential patients, especially in resource-limited health care settings.

In the present study, the total per patient cost of illness due to ISSI is 363.89 ± 86.32 USD which is nearly three times higher than the non-infective control group. (124.90 ±14.56USD). The value of COI obtained in our study may be different from the other studies. Such a difference in the value may be due to the type of cost included in the study, the type of surgeries, the country-specific differences in the health care settings, resources used, and economic environment of the countries. As in our case, the direct cost of treatment is fully borne by the government, and the prices included here are the actual material cost to the hospital which is obtained on subsided rates. However, the data obtained in our study coincides with the fact that overall there is 3 to 6 times higher spending on the patients of SSI compared to those who are not infected [4–6].

HAIs including SSI is one of the most preventable forms of infection. Such kind of cost of illness study is a useful indicator to give us an idea of how much extra society is spending on a condition. If the same efforts and resources are directed towards laying down and implementation of strict IPC practices, it will not only benefit us on the economic ground but can also help break the vicious cycle of further increasing the overexposure to high-end antimicrobial, length of hospital stay, and further development of resistance.

Our study has several limitations. Firstly, all participants were recruited from a single hospital setting, i.e. from a government-funded teaching hospital that mainly has patients from the rural population with a poor socio-economic background. Therefore, the results might not be generalizable to other health care settings with different regional and economic conditions.

Second, due to lack of resources, we have considered only ISSI patients, diagnosed during their inpatient stay or on follow-up visits. Active surveillance of all SSI patients was not carried out. Establishment of the nation or state-wide surveillance of SSI, with a large sample size, should be conducted to compare data obtained from various health care settings, which will provide a better understanding of the situation.

If the patient acquired an infection after her discharge and was treated at a different facility, such patients were not considered in the study.

Third, the intangible cost of treatment has not been considered in the study. Which reflects patients´ quality of life, including side-effects of pharmaceutical interventions and stress and anxiety, both caused by the SSI itself and keeping away the mother from the newborn baby. Although this has no direct financial impact, it is worth considering these factors. Furthermore, we can also take into account the intangible costs of the other family members including the sibling left alone without their mother for days.

## Conclusion

Development of SSI not only has a great economic impact with three times higher COI compared to the control group but also contributes to a significant increase in the use of high-end antimicrobials and duration of hospitalization and further increasing the risk of development of resistance.

## Supporting information

S1 Data (XLSX)
